# Dimensionality of the Wisconsin Schizotypy Scales-Brief Forms in College Students

**DOI:** 10.1155/2013/625247

**Published:** 2013-11-11

**Authors:** Eduardo Fonseca-Pedrero, Mercedes Paino, Javier Ortuño-Sierra, Serafín Lemos-Giráldez, José Muñiz

**Affiliations:** ^1^Department of Educational Sciences, University of La Rioja, C/Luis de Ulloa S/N, Edificio Vives, Logroño, 26002 La Rioja, Spain; ^2^Center for Biomedical Research in the Mental Health Network (CIBERSAM), Madrid, Spain; ^3^Department of Psychology, University of Oviedo, Oviedo, Spain

## Abstract

Wisconsin Schizotypy Scales are one of the most used measuring instruments for the assessment of psychometric risk for psychosis. The main goal of the present study was to analyze the internal structure of the Wisconsin Schizotypy Scales-Brief (WSS-B) forms and the reliability of the scores in a large sample of college students. The final sample was comprised by a total of 1349 students, 288 males, with a mean age of 20.48 years (SD = 2.58). The results indicated that the WSS-B scores presented adequate psychometric properties. Cronbach's alfa coefficient for total scores in WSS-B ranged from 0.86 to 0.93. Analysis of the internal structure of the WSS-B, through confirmatory factor analysis and exploratory structural equation modeling, yielded a four factor solution (Magical Ideation, Perceptual Aberration, Social Anhedonia, and Physical Anhedonia) as the most adequate. Statistically significant differences in mean scores of WSS-B by sex were found. These results provided new validity evidence of the WSS-B scores in an independent sample of nonclinical young adults. The WSS-B seems to be useful, brief, and easy to administrate for the screening of extended psychosis phenotype in the general population.

## 1. Introduction

The identification of individuals at risk for psychosis, whether in clinical or educational settings, requires having adequate measurement instruments that allow us to make solid and well-founded decisions based on the data. The main goal of the “psychometric high-risk” paradigm is the detection, by means of self-reports and/or interviews and based on their score profiles, of those participants with a higher theoretical risk of transiting toward a psychotic disorder in the future [1]. The “psychometric high-risk” paradigm is considered a reliable, valid, and useful method for the identification of individuals at risk for psychosis and its related disorders. The use of these tools constitutes, in comparison to other techniques, a rapid, efficient, and noninvasive method of assessment of the individuals at risk [2, 3]. Moreover, it allows the study of symptoms that are similar to those found in patients with psychosis while avoiding the confounding effects frequently found in these individuals (e.g., medication or stigmatization) [4].

There is a wide variety of measuring instruments for the assessment of schizotypy and extended psychosis phenotype [5], being the Wisconsin Schizotypy Scales (WSS) among the most widely used. Included in the WSS, we find the Perceptual Aberration Scale (PAS) [6], Magical Ideation Scale (MIS) [7], Revised Social Anhedonia Scale (RSAS) [8], and Revised Physical Anhedonia Scale (RPhA) [9]. The WSS scores have shown to be a vulnerability indicator in clinical samples [10] and a risk marker in nonclinical population [3, 11, 12]. Also, PAS and MIS scores have been directly predictive of conversion in adolescents at genetic high risk for psychosis [13]. Furthermore, WSS scores have shown relation with at risk mental states [14] and other psychopathological constructs (e.g., depression, anxiety) [15, 16]. Also, WSS scores have shown factorial equivalence across cultures [17], its ecological validity [18], and its psychometric properties are well established [5, 16]. When the internal structure underlying to WSS scores is examined, a two-dimensional structure—positive and negative dimensions—invariant across sex, age and culture have been found [16, 19]. Gender and age differences in the expression of the schizotypal phenotype has also been found. Using the Wisconsin Scales, males tend to score higher than females on the Negative dimension, or Anhedonia (RPhA, RSAS), whereas results in the positive dimension (PAS and MIS) are not still conclusive [20]. 

 Recently, Gross and collaborators have developed a brief version of WSS (WSS-B) [21, 22]. The administration of the WSS entails a long period of time (166 items). For this reason, the construction of an abbreviated version of the four WSS without loss of quality metric would be relevant and interesting from a clinical and research point of view. The selection of the final items that make up the WSS-B has been carried out rigorously and based on empirical criteria. Items depuration that composed the WSS-B has been done rigorously, being the metric properties analyzed from the Classical Test Theory and the Item Response Theory framework [23]. Differential item functioning was also examined for sex and ethnicity [23]. After items' purification, each of the four Scales was composed of 15 items. Those items high discrimination, and low differential item functioning were retained. Psychometric properties of WSS-B have been previously studied in college students samples [21, 22]; nevertheless, WSS-B have been recently developed and there are not preliminary data regarding internal structure and reliability of the scores. In this sense, it is interesting to conduct new studies to analyze the quality metric of the WSS-B in new samples that allow us to advance in its validation and to replicate previous findings. 

Within this research context, the main goal of the present study was to analyze the psychometric properties of the WSS-B scores in a sample of non-clinical young adults. With this aim, we examine the internal structure of the WSS-B, through confirmatory factor analysis and exploratory structural equation modeling, and we analyze the reliability of the scores. In addition, the influence of sex in the expression of WSS-B scores was examined. These goals would allow us to (a) deepen current knowledge regarding the psychometric characteristics of the WSS-B scores that can be better used for the detection of individuals at risk for psychosis in nonclinical populations; (b) improve the comprehension of schizotypy in a developmental stage of special risk for psychosis; and (c) advance in the field by further understanding the expression of the extended psychosis phenotype in non-clinical population.

## 2. Method

### 2.1. Participants

Participants came from two independent convenient samples of non-clinical population. Final sample was comprised by a total of 1349 college students (288 male; 21.35%). Mean age of the final sample was 20.48 (SD = 2.58), with a range of 17 to 32. Mean years of education was 17.1 (SD = 1.9). The first subsample was composed of 710 college students from different degree courses at the University of Oviedo (Education, Criminology, Psychology, Medicine, Speech Therapy, IT, Economics, and Physiotherapy). This first subsample was made up of 172 men (24.1%) and 539 women (75.9%). Mean age of the participants was 19.8 years (SD = 1.9), with a range of 17 to 27; mean years of education was 16.3 (SD = 1.9). Previous data of this sample have been used in other studies [19]. The second subsample was composed of a total of 639 college students from different degree courses at the University of Oviedo (Education, Psychology, Speech Therapy, Economics, and Physiotherapy) and University of La Rioja (Education). This second subsample was made up of 117 men (18.3%) and 522 women (81.7%). Mean age of the participants was 21.4 years (SD = 2.8), with a range of 17 to 30. Mean years of education were 18.1 (SD = 2.9). As regards marital status, 58.8% were single, 37.4% lived in couple, 2.7% were married, 0.3% were divorced, and 0.8% did not report their status. With regards to employment situation, 85.6% were not working and 14.4% were working. The 32.7% reported having a first-degree relative with antecedents of some other psychological disorder. 

### 2.2. Instruments

In the present work we used the Spanish WSS version adapted and validated in non-clinical young adults [19, 24, 25]. This adaptation was made in line with the international guidelines for test adaptation [26, 27]. *Magical Ideation Scale-Brief* (MIS-B) [7]: it is a self-report used for the assessment of superstitious and magical beliefs and thoughts as well as of the capacity of thought reading or broadcasting. It is composed of 15 items in a dichotomous True/False format.* Perceptual Aberration Scale-Brief* (PAS-B) [6]: the PAS-B has been used for the assessment of perceptual distortions associated with body image. It is composed of 15 items in a dichotomous True/False format.* Revised Physical Anhedonia Scale-Brief *(RPhA-B) [9]: the RPhA-B consists of 15 items in a True/False format, which measure the inability to experience pleasure from pleasant physical stimuli such as touching, smelling, or listening to music.* Revised Social Anhedonia Scale-Brief *(RSAS-B) [8]: the RSAS-B is composed of 15 items in a True/False format which measure schizoid indifference, associability, lack of social enjoyment, and indifference towards others. *Infrequency Scale* [28]: it consists of 13 items in a dichotomous True/False format (e.g., “*Driving from New York to San Francisco is generally faster than flying between these cities*”). The objective of the last scale is to detect those participants who respond randomly, pseudorandomly, or dishonestly to the measuring instruments; those subjects with 3 or more randomly answered items were eliminated from the final sample. 

### 2.3. Procedure

Administration of the measurement instruments was carried out in groups of 10 to 50 students, during normal lecture hours and in a room with appropriate conditions. The study was presented to the participants as a research project on diverse personality traits. It was stressed that their participation was voluntary and they were assured of the confidentiality of their responses. They received no type of incentive for taking part. Administration of the measurement instruments was always under the supervision of a researcher. This study is part of a broader research initiative on early detection and intervention in the context of psychological disorders in early adulthood and the analysis of psychopathological and personality variables.

### 2.4. Data Analysis

First of all, we calculated the descriptive statistics for the WSS-B. Second, we analyzed the internal structure of the WSS-B scores by means of confirmatory factorial analysis (CFA) and exploratory structural equation modeling (ESEM) conducted at item level [29–31]. The ESEM approach differs from the typical CFA approach in that all factor loadings are estimated, subject to constraints so that the model can be identified. Also, Structural Equation Modeling parameter estimates, standard errors, goodness-of-fit statistics, and statistical advances normally associated with CFA are reported. Here, we used an oblique Geomin rotation and the weighed least squares means and variance adjusted (WLSMV) estimator. The goodness-of-fit indices employed were the comparative fit index (CFI), the Tucker-Lewis index (TLI), the root mean square error of approximation (RMSEA), and standardized root mean square residual (SRMR). To achieve a good fit of the data to the model, the values of CFI and TLI should be over 0.95, and the RMSEA and SRMR values should be under 0.08 for a reasonable fit and under 0.05 for a good fit [32, 33]. Third, we estimated the reliability of the scores via Cronbach's alpha. In fourth place, with the aim to analyze the relation between mean scores of WSS-B and sex, a multivariate analysis of the variance (MANOVA) was conducted. As an index of size effect, eta partial square (partial *η* ^2^) was employed. For the data analysis we used SPSS 15.0 [34], FACTOR 9.2 [35], and Mplus 5.2 [31].

## 3. Results

### 3.1. Descriptive Statistics of the Scales and Estimation of Reliability of the WSS-B Scores

The descriptive statistics regarding mean, standard deviation, skewness and kurtosis, and maximum and minimum values for the WSS-B are shown in Table 1. Pearson's correlation coefficients between total scores of WSS-B are shown in Table 1. Correlations between total scores of the WSS and their brief version were calculated in the first subsample. Pearson's correlation coefficients between long and brief version were 0.91 for MIS, 0.90 for PAS, 0.83 for RPhA, and 0.80 for RSAS (*P* ≤ 0.01). Internal consistency level for the scores was 0.86 for MIS-B, 0.90 for PAS-B, 0.87 for RPhA-B, and 0.93 for RSAS-B.

### 3.2. Confirmatory Factor Analysis and Exploratory Structural Equation Modeling

Several confirmatory factor analyses were conducted testing different hypothetical models: (a) a one-dimensional model that could explain all the underlying symptoms to WSS-B scores; (b) a two-dimensional model, where MIS-B and PAS-B scores are grouped into a positive factor and RPhA-B and RSAS-B scores into a negative factor; (c) a model with four dimensions (Magical Ideation, Perceptual Aberration, Social Anhedonia and Physical Anhedonia); (d) a model with a general second order factor of schizotypy and four first order factors (Magical Ideation, Perceptual Aberration, Social Anhedonia, and Physical Anhedonia); and (e) a model with two second order factors (positive and negative) and four first order factors (Magical Ideation, Perceptual Aberration, Social Anhedonia, and Physical Anhedonia). Goodness-of-fit indices for the hypothetical models tested are shown in Table 2. As it is presented, the four factor model showed the best goodness-of-fit indices in comparison with the other models tested. The model with two second order factors and four first order schizotypy factors was impossible to test due to a problem with the latent PSI variable of the covariance matrix that was not positive definite.

In addition, in the frame of ESEM, three schizotypy dimensional models were tested. Goodness-of-fit indices for proposed models of one, two, and four schizotypy factors are shown in Table 2. Four factor model displayed the best goodness-of-fit indices. Standardized factor loadings for this dimensional model are shown in Table 3. First factor grouped items related to Magical Ideation. Second factor grouped items related to Perceptual Aberration. Third factor grouped items related to Physical Anhedonia. Fourth factor grouped items related to Social Anhedonia. Only a few number of items were not related to their correspondent dimension. As it is shown, several items of Magical Ideation factor showed cross-loadings in factor II Perceptual Aberration, indicating some overlap between both dimensions. For this hypothetical solution of four schizotypy factors estimated through ESEM, correlation between latent factors ranged between 0.41 (FI-FII) and −0.08 (FI-FIV) (*P* < 0.01).

### 3.3. Mean Scores Comparisons in WSS-B according to Sex

Wilk's *λ* value revealed statistically significant differences by sex (*λ* = 0.941, *F*_(4,1344)_ = 21.044, *P* ≤ 0.001, *η* ^2^  partial = 0.059). Mean scores and standard deviation by sex in the four WSS-B are shown in Table 4. Statistically significant mean scores differences were found in the Physical and Social Anhedonia, where men showed higher mean scores than women. 

## 4. Discussion and Conclusions

The main goal of this work was to study the psychometric properties of the Wisconsin Schizotypy Scales-Brief forms (WSS-B) [21, 22] in a large sample of non-clinical young adults. For this purpose, we examine the internal structure of the WSS-B scores, through confirmatory factor analysis (CFA) and exploratory structural equation modeling (ESEM), and we analyze the reliability of the scores. In addition, we examine the influence of sex in the WSS-B. The results indicate that the WSS-B are brief measurement instruments, with adequate psychometric properties for the assessment of extended psychosis phenotype, and that could be used as screening tools for detection of individuals at risk for psychosis in the general population. 

Analysis of the internal structure through CFA and ESEM showed that the hypothetical model with four schizotypy factors (Magical Ideation, Perceptual Aberration, Physical Anhedonia, and Social Anhedonia) yielded the best goodness-of-fit indices. Items related in these four factors did not show high cross-loadings and factor loadings estimated showed high weight. It is noteworthy that dimensional models tested here were complex, due to the high number of items and the high overlap in the item content. Moreover, it is very interesting to test second order dimensional models (where items are grouped in first order factors and at the same time in second-order general schizotypy dimensions) that allow us to capture with more clarity the complexity and the heterogeneity of the underlying structure of the WSS-B scores. Moreover, these new dimensional models tested permit to improve the comprehension of schizotypy construct and psychosis phenotype. 

It is known that schizotypy is a multidimensional construct similar to that found in patients with schizophrenia [1, 36]. Previous studies have analyzed the dimensional structure of the WSS in samples of college students [16, 17, 19] showing the presence of two schizotypy dimensions (positive and negative) where social anhedonia is related to both factors. In the present study the model with two second order factors and four first order factors was not computed, reason why it is not possible to check its possible relation with the model proposed for Kwapil et al. [16]. Even though the comparison between factorial studies about schizotypy is hampered for the heterogeneity of the samples, measure instruments, and statistical techniques used, results revealed that positive and negative schizotypy dimensions are the most replicated [37–40]. Due to the fact that this study presents the first analysis of the internal structure of the WSS-B conducted at item level, future studies should try to replicate these findings and could test different hypothetical dimensional models (e.g., social anhedonia grouped in schizotypy positive factor).

Cronbach's alfa coefficient for total scores ranged to 0.86 from 0.93. Alfa values are appropriate and reveal that the instruments measure accurately the schizotypy construct. Previous studies have found similar reliability values. For example, Gross et al. [21] in two large samples of college students (*n* = 6137, *n* = 2171) found that reliability levels for WSS-B ranged between 0.86 and 0.95 (binary alpha) and between 0.86 and 0.94 (binary alpha), respectively. These data suggest that short forms WSS, compared to the original WSS, continue with similar reliability levels [16, 21]. The WSS-B scores showed a differential pattern by sex. Men showed higher mean scores than women in Physical and Social Anhedonia; nevertheless, we did not find statistically significant differences in Magical Ideation and Perceptual Aberration facets. Previous research conducted with WSS original versions found similar results [16, 40, 41]. For instance, in a meta-analysis conducted by Miettunen and Jääskeläinen [20] they found that men presented higher scores than women in Anhedonia, whereas differences were not found in schizotypy positive dimension. However, it should be pointed out that other studies have indeed found higher scores in females for the positive dimension [16]. These results are similar to other studies where schizotypy is analysed with other self-reports (e.g., O-LIFE) [37] and in non-clinical adolescent population [42, 43]. 

Data found in this work are preliminary and show new validity evidence based on the internal structure and reliability for WSS-B scores. Nevertheless, these results should be interpreted in the light of the following limitations. First of all, the sample characteristics (college students and predominantly women) preclude the generalization of the results to other populations of interest. Second, given the problems inherent in any type of study based on self-reports, it would have been useful to employ reports from external informants. Finally, it should be borne in mind that this study was of a cross-sectional nature, so that we cannot make cause-effect inferences. For this reason, it is necessary to keep on the examination of the metric properties and to replicate these findings in future studies.

Other future studies should examine the psychometric properties of the WSS-B in other samples (e.g., adolescents) and high-risk paradigms (e.g., ultra high risk) [44, 45]. Likewise, it would be interesting to incorporate a response format taking into consideration the preoccupation, conviction, and associated distress [46]. Finally, it would be interesting to introduce schizotypy studies into the Research Domain Criteria Framework [47].

## Figures and Tables

**Table 1:** Descriptive statistics and Pearson's correlation coefficients for the short forms of the Wisconsin Schizotypy Scales.

	M	SD	Skweness	Kurtosis	Min, max	MIS-B	PAS-B	RSAS-B	RPhA-B
MIS-B	2.08	2.22	1.34	1.63	0–12	1			
PAS-B	1.12	1.88	2.24	5.68	0–13	0.51*	1		
RSAS-B	0.95	1.62	3.14	13.31	0–13	0.10*	0.11*	1	
RPhA-B	2.43	2.20	1.22	1.62	0–13	−0.05	0.01	0.21*	1

*P < 0.01.

Note: MIS-B: Magical Ideation Scale-Brief; PAS-B: Perceptual Aberration Scale-Brief; RSAS-B: Revised Social Anhedonia Scale-Brief: RPhA-B: Revised Physical Anhedonia Scale-Brief.

**Table 2:** Goodness-of-fit indices of hypothetical models tested.

Model	*χ* ^2^	df	CFI	TLI	RMSEA	SRMR
CFA						
1 factor	1505.42	252	0.514	0.560	0.061	2.226
2 factors	768.66	259	0.804	0.827	0.038	1.572
4 factors	572.95	264	0.880	0.896	0.030	1.340
1 general factor + 4 first order factors	630.47	250	0.853	0.865	0.034	1.457
ESEM						
1 factor	1505.42	252	0.514	0.560	0.061	2.226
2 factors	846.07	292	0.785	0.832	0.038	1.440
4 factors	570.19	317	0.902	0.929	0.024	1.034

Note: CFA: confirmatory factor analysis; ESEM: exploratory structural equation modeling; *χ* ^2^: chi square; df: degrees of freedom; CFI: comparative fit index; TLI: Tucker-Lewis index; RMSEA: root mean square error of approximation; SRMR: standardized root mean square residual.

**Table 3:** Estimated factorial loadings for the four factor model through exploratory structural equation modeling.

Items	FI	FII	FIII	FIV
1	**0.300 **	**0.508 **	−0.244	0.077
2	**0.300 **	**0.497 **	−**0.308**	0.123
3	**0.345 **	0.232	0.020	−0.044
4	**0.416 **	**0.333 **	−0.139	−0.075
5	0.000	**0.365 **	0.121	−0.301
6	**0.305 **	**0.494 **	−0.061	0.035
7	0.107	**0.491 **	0.075	−0.267
8	0.189	**0.329 **	0.049	−0.077
9	**0.321 **	**0.317 **	0.009	−0.127
10	0.189	**0.355 **	0.041	−0.164
11	0.074	0.284	−0.045	−0.023
12	0.275	**0.413 **	0.156	−0.113
13	0.140	**0.447 **	0.071	−0.254
14	−0.028	**0.478 **	−0.061	−0.159
15	0.129	**0.432 **	0.127	−0.120
16	**0.556 **	0.190	0.126	−0.153
17	**0.864 **	−0.434	0.110	−0.083
18	**0.777 **	−0.121	−0.216	0.137
19	**0.776 **	−0.034	0.085	−0.035
20	**0.817 **	−0.013	−0.076	0.142
21	**0.546 **	0.233	−0.226	0.016
22	**0.814 **	0.009	−0.015	0.054
23	**0.548 **	0.074	0.155	−0.221
24	**0.800 **	−0.230	−0.220	0.018
25	**0.576 **	0.144	0.126	0.004
26	**0.571 **	0.186	−0.196	0.110
27	**0.389 **	0.237	0.092	−0.088
28	**0.566 **	0.262	−0.017	0.064
29	**0.687 **	0.051	0.025	−0.002
30	**0.647 **	0.239	0.035	−0.161
31	−0.098	−0.033	0.001	0.134
32	0.135	0.095	0.193	**0.430**
33	−0.039	0.000	0.260	**0.374**
34	0.011	−0.029	**0.330**	**0.511**
35	−0.045	0.286	0.080	**0.764**
36	−0.052	0.068	0.028	**0.479**
37	−0.073	0.098	0.176	**0.147**
38	0.039	−0.073	0.247	**0.671**
39	0.068	−0.172	0.285	**0.488**
40	−0.067	0.205	−0.052	**0.745**
41	0.088	0.048	0.109	**0.371**
42	−0.009	−0.059	0.285	**0.289**
43	0.072	−0.127	0.067	**0.376**
44	0.017	0.072	0.157	**0.488**
45	−0.010	0.280	−0.024	**0.742**
46	−0.059	0.106	**0.813**	−0.010
47	0.040	−0.109	**0.681**	0.044
48	0.083	−0.020	**0.743**	0.136
49	−0.024	0.191	**0.574**	0.124
50	0.014	−0.048	**0.875**	0.144
51	0.054	0.107	**0.701**	−0.057
52	−0.093	−0.111	**0.863**	0.029
53	0.068	0.052	0.293	0.160
54	0.036	−0.027	**0.570**	**0.303**
55	−0.002	0.208	**0.505**	0.018
56	0.058	0.132	**0.666**	−0.043
57	0.016	0.276	**0.485**	0.168
58	−0.035	**0.404**	**0.466**	0.130
59	0.023	−0.043	**0.648**	−0.082
60	−0.058	0.095	**0.511**	−0.075

Note: items 1–15 belong to Magic Ideation; items from 16 to 30 belong to Perceptual Aberration; items 31–45 belong to Physical Anhedonia; and items 46–60 belong to Social Anhedonia. Items with factorial loadings greater than 0.30 are shown in bold.

**Table 4:** Mean scores comparisons in the Wisconsin Schizotypy Scales-Brief by sex.

Score	Men		Women				
	M	SD	M	SD	*F*	*P*	*η* ^2^ partial
MIS-B	2.28	2.30	2.02	2.20	3.135	0.077	0.002
PAS-B	1.18	1.81	1.10	1.89	0.356	0.551	0.000
RSAS-B	1.28	2.01	0.87	1.48	15.023	≤0.001	0.011
RPhA-B	3.40	2.70	2.17	1.96	74.468	≤0.001	0.052

Note: MIS-B: Magical Ideation Scale-Brief; PAS-B: Perceptual Aberration Scale-Brief; RSAS-B: Revised Social Anhedonia Scale-Brief: RPhA-B: Revised Physical Anhedonia Scale-Brief.
